# The Template-Jumping Editing Approach in *F9*-Associated Hemophilia B Gene Therapy

**DOI:** 10.3390/ijms262411916

**Published:** 2025-12-10

**Authors:** Robert Sattarov, Alexey Kuznetsov, Valeriy Klimko, Elena Ignatyeva, Roman Ivanov, Alexander Karabelsky, Anastasia Fizikova

**Affiliations:** Translational Medicine Research Center, Sirius University of Science and Technology, 1 Olympic Ave., Federal Territory Sirius, 354340 Sirius, Russia; sattarov.rf@talantiuspeh.ru (R.S.);

**Keywords:** hemophilia B, coagulation factor IX, *F9*, CRISPR, Cas, prime editing, gene therapy

## Abstract

Hemophilia B is a hereditary bleeding disorder caused by mutations localized throughout the *F9* gene. Existing gene therapy products containing AAV vectors have significant limitations. Replacement therapy with coagulation factor FIX infusions is not an optimal way of treatment, as patients still have periodic bleeding and require frequent transfusions. Moreover, approximately 5% of adult patients with hemophilia B develop inhibitory antibodies to recombinant forms of FIX. Therefore, it is important to develop universal CRISPR/Cas gene therapy approaches for *F9* editing using non-viral delivery systems to enable gene reversion to a functional sequence at an early stage of disease development and establishment of the patients’ immune system. In this study, a unique approach of *F9* prime-editing was tested for the first time. This method is estimated to edit 7.3% of pathogenic *F9* mutation types. Specifically, it targets the gene region encoding amino acids 374 V to 408 Q, which accounts for approximately 9.35% of patients with hemophilia B. An advantage of this gene therapy approach is the absence of the need to change Primer Binding Site (PBS) or Reverse Transcriptase Template (RTT) sequences until going from preclinical to clinical trials, as well as the introduction of gain of function mutations in order to compensate for the low prime-editing frequencies and enhance the effect of treatment in vivo.

## 1. Introduction

Hemophilia B is one of the most well-known hereditary human diseases, characterized by a monogenic X-linked inheritance. Current approaches for delivering gene therapeutic products via adeno-associated virus (AAV) vectors have significant limitations: in actively dividing cells, the episomal vector is gradually lost, along with the associated therapeutic effect. Therefore, these approaches are more suitable for adult patients whose hepatocytes are no longer characterized by frequent divisions. In adult patients with hemophilia B, approximately 5% develop inhibitory antibodies against recombinant forms of factor IX (FIX) [1]. This highlights the importance of developing universal gene therapeutic approaches using CRISPR/Cas editing of *F9*, using non-viral delivery systems to enable the reversion of the gene to a functional sequence at an early stage of disease development and during the maturation of the patients’ immune systems. The *F9* gene is located near the long arm of the human X chromosome, spans 34 kilobases, and is the largest gene among those encoding vitamin K-dependent coagulation factors. The spectrum of mutations leading to Hemophilia B is extremely diverse, encompassing all possible types of mutations, which are evenly distributed across both the coding sequence and the regulatory and intronic regions (for example, Leiden disease arises from a mutation in the promoter region of *F9*). Notably, there are no so-called “hot spot” regions for mutations in *F9*. Furthermore, while documenting one mutation in a patient, other minor mutations may also be observed upon deeper sequencing of *F9* sequences [2,3].

Genome editing using CRISPR/Cas technologies represents a revolutionary advancement in molecular biology and genetic engineering. Genome editing methods that use components of the adaptive immune system of bacteria and archaea allow targeted alterations of DNA in cells, opening new horizons for the treatment of heritable diseases. The development of CRISPR/Cas gene therapeutic approaches began in 2013 [4,5], yet no effective universal strategies for achieving high rates of biallelic reversion of mutations have been developed, both in cell lines and in vivo, which can be attributed to the low frequency of homology-directed repair (HDR) in mammalian cells [6,7]. The primary mechanism for repairing double-strand breaks in mammals is non-homologous end joining (NHEJ). The main mechanism of CRISPR/Cas action involves the use of guide RNA (gRNA), which binds complementarily to the target DNA, allowing the Cas9 nuclease to cleave the DNA. Following the DNA cleavage, repair mechanisms such as NHEJ and homologous recombination (HR) are activated in the cells. NHEJ is a rapid but less accurate mechanism that can lead to insertions or deletions (indels) at the cleavage site. Conversely, HR requires the presence of a DNA template and provides more precise repair and restoration, allowing for targeted changes to be made to the genomic sequence.

Prime editing is a genome editing technology that employs a combination of gRNA and reverse transcriptase to introduce changes into the DNA sequence without creating double-strand breaks, using a fusion of catalytically altered Cas9 (or nickase) and the reverse transcriptase of Moloney murine leukemia virus (M-MLV) with gRNA (pegRNA). The pegRNA directs the target DNA and facilitates the synthesis of a complementary strand with the desired modifications. The pegRNA consists of an 18–20-nucleotide spacer sequence that instructs Cas9 on which DNA locus to modify, PBS, and RTT). The PBS sequence binds to the single-stranded DNA (ssDNA) of the genome after it has been cleaved and acts as a primer for the initiation of complementary DNA strand synthesis. The RTT functions as a template for the reverse transcriptase to synthesize the necessary complementary strand with the desired modifications. The most valuable aspect of the relatively new technology of prime editing is its ability to correct up to 89% of known genetic diseases due to more precise and versatile editing capabilities compared to traditional CRISPR/Cas9 methods [8]. However, the limitations of prime editing include low efficiency for large insertions (>400 base pairs) and restricted applicability in vivo. Template-jumping is a prime editing method based on the mechanism of retrotransposition, allowing for the insertion of large (up to 800 base pairs) DNA fragments using overlapping gRNAs (pegRNAs) and nicking gRNAs [9]. The drawbacks of this method include a low editing frequency, which has been improved by introducing the negative regulator of mismatch repair, MLH1, into the editing system, or by employing peptides that bind intracellular MLH1 to the prime editor. This modification results in an 18-fold increase in editing efficiency [10,11]. However, such an approach carries the risks of inducing carcinogenesis when employed in vivo [12]; therefore, it is crucial to explore alternative methods for influencing the repair mechanisms of the edited cell. Nevertheless, the template-jumping approach to genome editing still holds significant potential for the treatment of hereditary monogenic diseases, such as hemophilia B. The aim of this study is to evaluate the prime-editing approach based on the mechanism of retrotransposition and to compare it with classical prime editing using a model of hemophilia B.

## 2. Results

### 2.1. Human Hepatocyte Cell Line with F9 Gene Knockout

**Figure 1:**
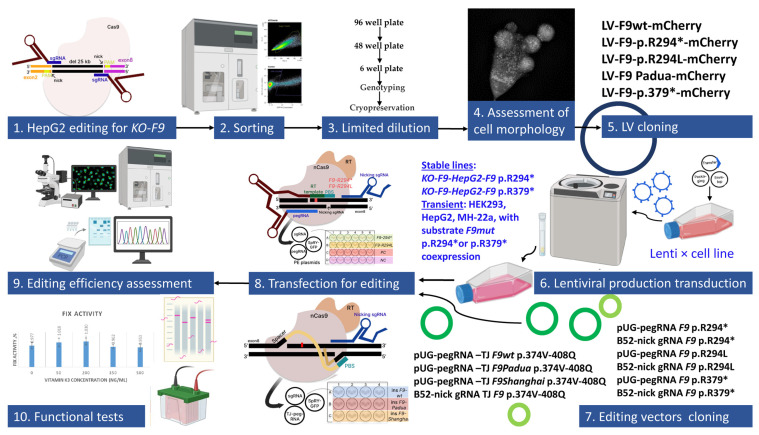
Schematic overview of experiments: nonsense mutations are marked with an asterisk.

In the initial stage of our work, we aimed to assess the efficiency and accuracy of the prime editing approaches used for reverting mutant alleles of the *F9* gene and reducing the likelihood of off-target editing, as well as allowing for a more precise evaluation of changes in the concentration and activity of FIX (Figure 1).

**Figure 2:**
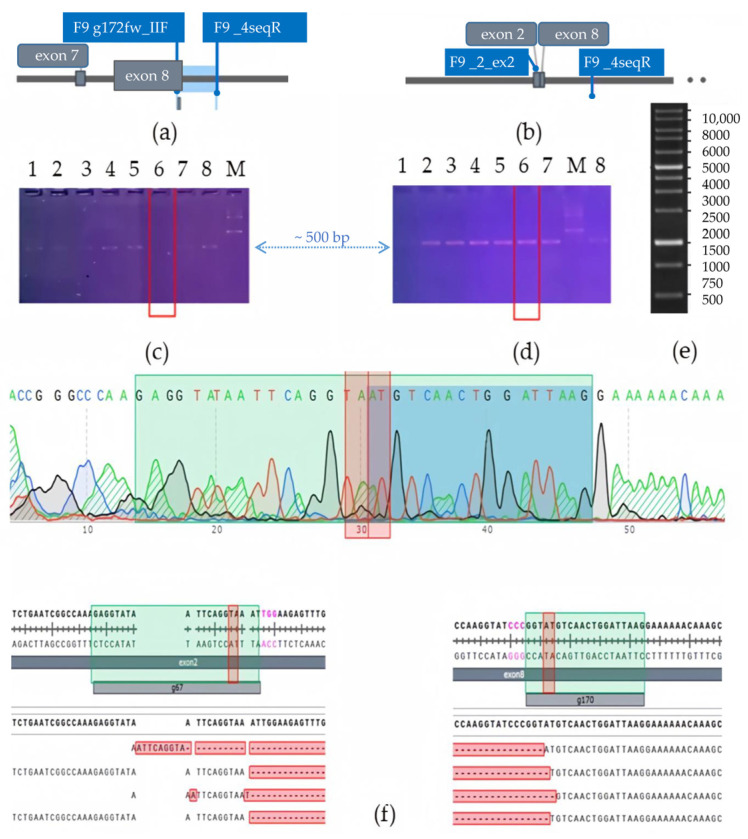
Genotyping of HepG2 cells for selection of KO-*F9* clones: (**a**) Schematic representation of the localization of the complementary *F9* g172fw_IIF and *F9*_4seqR primers targeting regions of the *F9* gene; (**b**) Schematic representation of the localization of the complementary *F9*_ex2_F and F9_4seqR primers targeting regions of the *F9* gene: in the case of successful CRISPR/Cas9 editing resulting in a deletion of 25,000 base pairs of the *F9* gene and the proximity of exons 2 and 8, a PCR product of 539 base pairs will be amplified; (**c**) Electrophoresis of PCR products using F9 g172fw_IIF and *F9*_4seqR primers: PCR products for detection of F9wt alleles in the HepG2 pool are presented from left to right (1–8), M–1 kb DNA marker (Thermo Fisher); (**d**) Electrophoresis of PCR products using *F9*_ex2_F and *F9*_4seqR primers: PCR products for detection of deleted *F9* alleles in the pool are presented from left to right (1–7,8), M–1 kb DNA marker (Thermo Fisher): red line marks the lanes with PCR products of the clone containing an extended deletion of the *F9* gene; the blue arrow marks the 500 base pair level; (**e**) Gene Ruler 1 Kb DNA Ladder scheme; in electrophoresis images (**c**,**d**), PCR products amplified from the same genomic DNA are shown in the order of loading: the sample not containing cells with the wild type *F9* allele is marked with a red box; a 1% agarose gel, TAE buffer, and a 1kb DNA fragment size marker (Thermo Fisher) were used for both separations. (**f**) Sanger sequencing of the amplicon flanked by the sequences of the primers *F9*_ex2_F and *F9*_4seqR: the chromatogram of the amplicon is displayed at the top, with green rectangles indicating the regions of gRNA g67 (left) and g170 (right), and red rectangles marking 3–4 nucleotides from the PAM (marked in pink), where double-strand breaks in the DNA are most likely hydrolyzed by the Cas9 nuclease. The results of decomposition the obtained sequence traces in the online tool ICE (EditCo) are presented at the bottom of the figure, showing that the amplicon contains large deletions of the *F9* gene.

Thus, we generated cell lines with a large deletion of the *F9* gene. A combination of gRNAs complementary to exons 2 and 8 of the *F9* gene was selected for this purpose. Successful knockout resulted in a deletion of 25,000 base pairs, excising the region of the gene that encodes nearly the entire protein: from the GLA domain (K51) to the end of the protease domain (Y450). In total, a limiting dilution of 10 different populations of HepG2 cells was performed following editing and sorting, resulting in the selection of two populations where genotyping indicated a predominance of the *F9* gene variant with the extended deletion. During genotyping, two edited polyclonal HepG2 cell lines were identified, which contained a deletion spanning from exon 2 to exon 8 of the *F9* gene in the majority of the cell pool (Figure 2).

**Figure 3:**
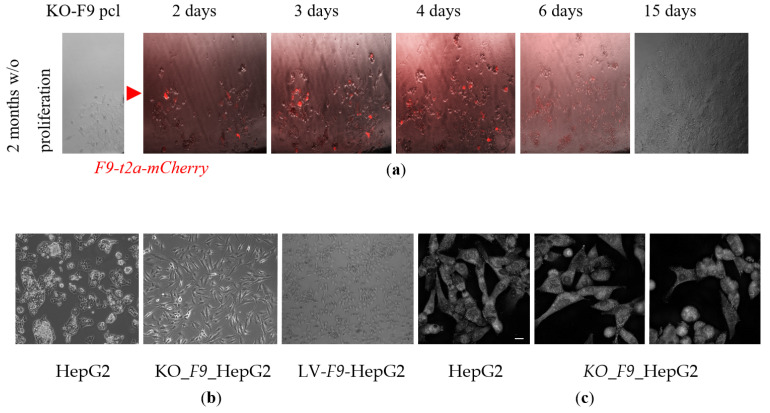
The impact of *F9* knockout and overexpression on the morphology of HepG2 cells: (**a**) The proliferation dynamics of the KO-*F9*-HepG2 polyclonal line over a 15-day period following transfection with a lentiviral vector containing the *F9*-t2a-mCherry construct for transient expression of F9wt (bright field and fluorescent microscopy, 10× magnification). The red arrow indicates the stage of transfection with the lentiviral vector for transient expression of F9wt; (**b**) Morphology of HepG2 lines observed through phase-contrast microscopy at 60% confluence, arranged from left to right: HepG2 in a T-25 flask; KO_*F9*_HepG2 in a 6-well plate; HepG2-LV-*F9*-WT in a T-25 flask (10× magnification); (**c**) Morphology of HepG2 lines visualized using holotomography (20 µm scale bar).

Following the initial genotyping, the KO_F9_HepG2 population exhibited slow proliferation and a prolonged adaptation period. After a period of one to two months, the cells began to proliferate; however, genotyping revealed a redistribution of the cell population composition, with an increasing prevalence of cells exhibiting the (g172fw_IIF and F9_4seqR) PCR-product^+^, characteristic of cells without large 25 kb deletion of *F9*. This pattern was consistently observed on the two selected CRISPR/Cas9 edited subpopulations, which were selected through flow cytometry. To determine whether the observed redistribution of the edited cell population was a consequence of proliferation arrest due to the extended deletion of the *F9* gene or a selective advantage of residual cells with wild type *F9* alleles in the selected populations, the genotyped and characterized population of edited cells, predominantly containing KO-*F9* alleles, was subjected to extensive dilution for the selection of a monoclonal line. Colonies that were stagnant and did not proliferate for a month were then transfected with a lentiviral vector containing *F9wt*-mCherry (Figure 3)

On the sixth day of *F9* expression, many cells developed pro-apoptotic blebs, lost adhesion, and acquired a rounded morphology. After approximately two weeks, the remaining cells adhered to the plate proliferated and reached 90% confluence (Figure 3a). Upon reaching 80–90% confluence, the KO-*F9*-HepG2 polyclonal line was genotyped: the surviving cell pool retained alleles with varying lengths of *F9* deletions, but the majority of cells contained the wild type *F9* allele, as confirmed by Sanger sequencing. Thus, following the induction of proliferation, the proliferative advantage was observed in cells with the wild type *F9* allele in the polyclonal edited KO-*F9*-HepG2 line. Different growth stages of the cells were distinctly manifested in their varied morphology when plated simultaneously: the parental HepG2 line exhibited greater confluence, with cells interconnected and lacking prominent protrusions, growing upwards rather than spreading across the surface, whereas KO-*F9*-HepG2 displayed lower confluence, with some cells exhibiting pronounced protrusions characteristic of proliferating cells, while others lost adhesion to the plate surface and assumed a round shape (Figure 3b,c).

In most published studies aimed at developing FIX or gene therapy approaches, the immortalized HepG2 hepatocyte line is employed without additional modifications, with high levels of expression achieved through lentiviral transduction or gene delivery using AAV [13,14]. Therefore, the next stage of our work involved the establishment of stable lines using lentiviral transduction for the subsequent evaluation of the efficiency of prime editing.

### 2.2. Stable Lines with Substrate Chimeric Mutant Sequences F9-t2a-mCherry for Testing of Prime Editing Efficiency

To assess editing frequencies in both transient models and stable cell lines, we constructed lentiviral vectors containing chimeric sequences of the human *F9* gene with nonsense mutations c.880C > T, p.R294* and c.1135C > T, p.R379*. Successful editing of the substrate sequence with nonsense mutations leads to the restoration of the reading frame of the gene, resulting in the formation of the chimeric transcript *F9-t2A-mCherry*, cotranslational cleavage of the t2A peptide, and the folding of equimolar amounts of FIX and mCherry proteins, phenotypically manifested as the presence of mCherry fluorescence.

The KO_*F9*-HepG2 cells were transfected with a mix of editing vectors aimed at reverting the *F9* mutations in a 24-well format, with 5 technical and 4 biological replicates. Three vector mixtures were used according to the PE3 strategy. Control transfections in this group of experiments were conducted using the vector LV-*F9wt*-mCherry in equal mass quantities to the editing-target vector LV-*F9 (*p.R294*)-mCherry. Therefore, in these experiments, the smaller the observed difference in the number of mCherry^+^ cells between the control and experimental samples, the higher the efficiency of the prime editing performed.

**Figure 4:**
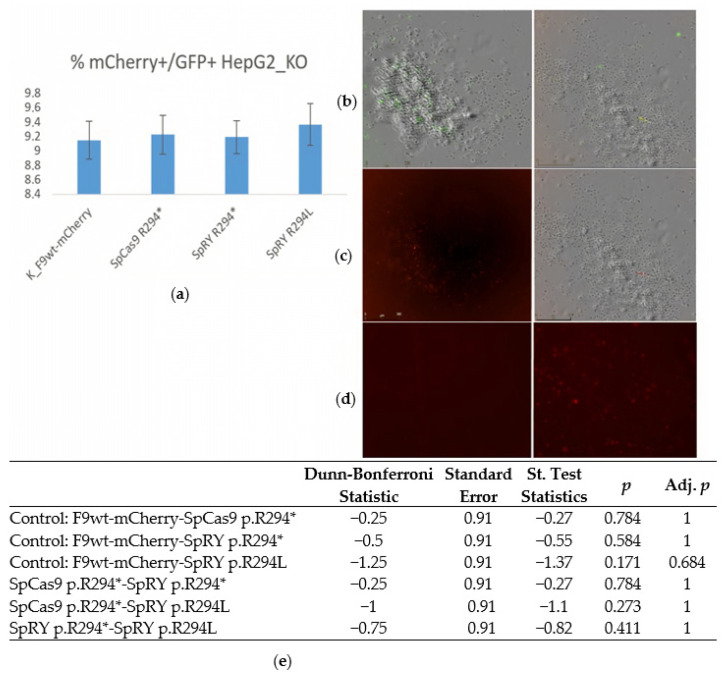
Evaluation of editing frequency of *F9*(p.R294*)-mCherry during transient expression in HepG2_KO_*F9* Cells: (**a**) A histogram displaying the proportions of GFP^+^mCherry^+^ HepG2_KO-*F9* cells transfected with prime-editing constructs: SpCas9 p.R294*: pU6-pegRNA-*F9*(p.R294*)-SpCas9, pSpCas9-GFP, nicking gRNA_*F9*(p.R294*), LV-*F9*(p.R294*)-mCherry; SpRY p.R294*: pU6-pegRNA-*F9*(p.R294*)-SpRY, SpRY-GFP, nicking gRNA_*F9*(p.R294*), LV-*F9*(p.R294*)-mCherry; SpRY p.R294L: pU6-pegRNA-*F9*(p.R294L)-SpRY, SpRY-GFP, nicking gRNA_*F9*(p.R294*), LV-*F9*(p.R294*)-mCherry (Incucyte microscopy). The Friedman test indicated that there was no statistically significant difference among the dependent variables, *p* = 0.552 (Chi^2^ = 2.1; df = 3). To further confirm the absence of differences in the proportions of GFP^+^mCherry^+^ cells between the control and experimental groups, the Dunn–Bonferroni post hoc test was applied. (**b**) HepG2_KO-*F9* cells 48 h post-transfection with editing vectors, showing an overlay of bright-field and GFP fluorescence microscopy, scale bar 500 µm. (**c**) HepG2_KO-*F9* cells 48 h post-transfection with editing vectors, mCherry fluorescence, scale bar 500 µm; (**d**) Left, *F9*wt-mCherry control cells on the right: mCherry fluorescence, scale bar 500 µm; (**e**) Table of results from pairwise comparisons using the Dunn-Bonferroni test of mCherry+ cell frequencies upon transfection with lentiviral vectors *F9*-t2a-mCherry as a control and edited pools of cells with substrate vectors containing nonsense and missense mutations in *F9*: the absence of differences in mCherry+ frequencies between control cells and edited transient model cells with substrate vectors containing nonsense mutations in *F9* indicates a high frequency of restoration of the reading frame of the substrate sequences.

Forty-eight hours post-transfection, the fluorescence of the reporters was analyzed using a live-cell imaging system (Figure 4).

**Figure 5:**
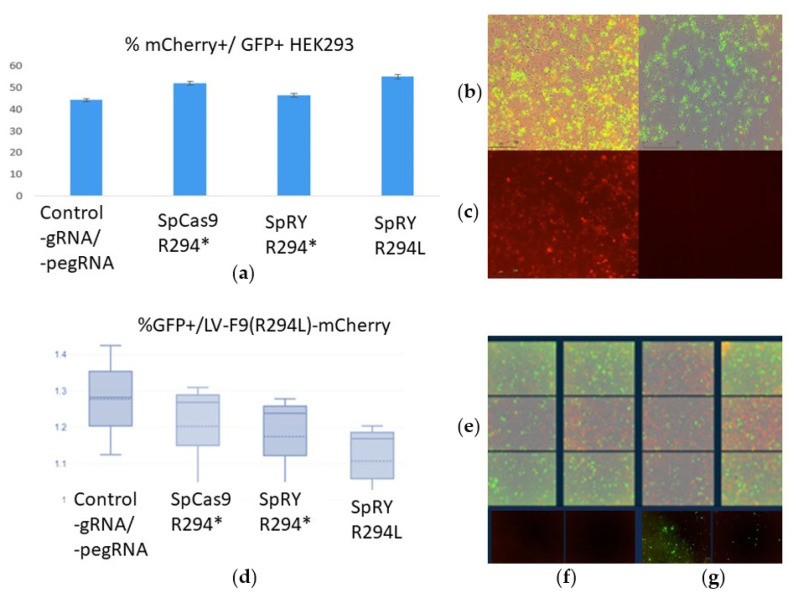
The efficiency of transfection and prime editing in HEK293 and HepG2 lines: (**a**) Transfection efficiency (GFP^+^) and prime editing of the LV-F9-R294*-mCherry sequence: in the case of successful editing, the reading frame is restored, and mCherry begins to be expressed; (**b**) Transfected HEK293 cells with editing vectors, overlay of bright field and fluorescence microscopy in two channels (mCherry and GFP) on the left; overlay of bright field and GFP on the right, scale bar 200 µm; (**c**) Transfected HEK293 cells with F9wt-mCherry control cells without any pegRNA on the left; Transfected control HEK293 cells (PE without any pegRNA) on the right, scale bar 200 µm; (**d**) Transfected HEK293 cells with editing vectors, mCherry fluorescence; (**d**) Transfection efficiency (GFP^+^) of HepG2/LV-*F9*-R294L-mCherry editing vectors, medians, means, and standard deviations are shown; (**e**) Transfected HepG2/LV-*F9*-R294L-mCherry cells with editing vectors (24 fields of view detectable by Incucyte), overlay of bright field and fluorescence microscopy in two channels (mCherry and GFP); (**f**) Transfected control HepG2 cells (PE without any pegRNA), mCherry fluorescence, scale bar 200 µm; (**g**) Transfected control HepG2 cells (PE without any pegRNA), GFP fluorescence, scale bar 200 µm.

The figure illustrates that upon transfection with the vector LV-*F9*(p.R294*)-mCherry and three mixtures of editing vectors, the number of GFP^+^mCherry^+^ HepG2_KO-*F9* cells observed was statistically indistinguishable from the control. In the control transfections, the presence of mCherry fluorescence was attributed to the LV-*F9wt*-mCherry construct. In contrast, the appearance of mCherry fluorescence in the experimental samples could only be due to the restoration of the reading frame and successful prime editing of the LV-*F9*(p.R294*)-mCherry sequence, which contained a nonsense mutation within the *F9* sequence. The efficiency of repair and prime editing of target sequences may vary between transient expression within lentiviral vectors and the integration of target sequences into the genome during transduction. Therefore, next, we obtained HepG2 cells transduced with lentiviruses containing integrated sequences *F9*-(p.R294L)-mCherry, *F9*-(p.R294*)-mCherry, and *F9*-(p.R379*)-mCherry. The cells were then transfected with mixes of prime-editing vectors. The controls for this experiments included transfections with LeGO-g vectors in equal mass quantities of DNA as in the experimental samples, as well as transfections of the HEK293 cell line (Figure 5).

During the transfection of HEK293 cells, the control transfections used the LV-*F9wt*-mCherry vector in equal mass quantities as the vector with the target for editing sequence LV-*F9*(p.R294*)-mCherry. Therefore, in the experiments with HEK293, the smaller the observed difference in the number of mCherry^+^ cells between the control and experimental samples, the higher the efficiency of the prime editing performed. Observing the dynamics of the accumulation of fluorescent signals from GFP and mCherry in HEK293 cells revealed that mCherry fluorescence accumulates gradually and is observed in a large number of GFP^+^ HEK293 cells, indicating successful restoration of the reading frame in the LV-*F9*-p.R294*-mCherry construct (Supplementary Videos S1 and S2).

### 2.3. Prime Editing of the p.R379* Mutation in the F9 Gene via Retrotransposition Mechanism in a Transient Model on the HEK293 Cell Line

**Figure 6:**
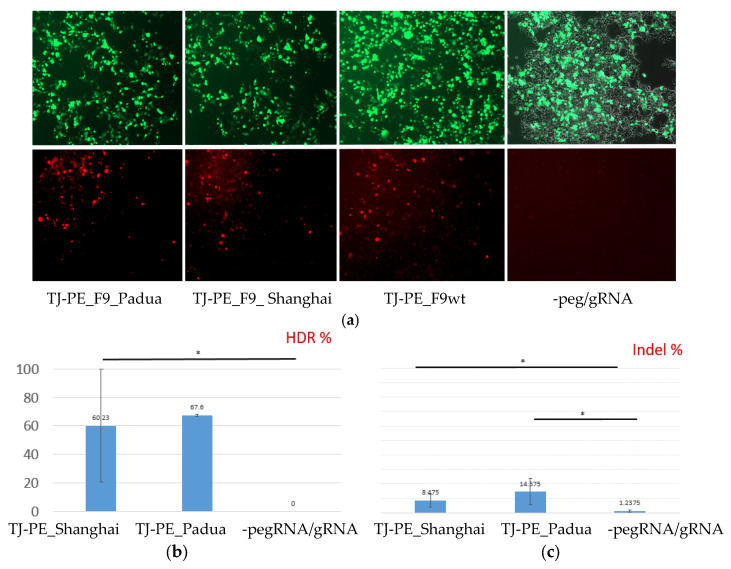
Editing of the *F9* p.R379* mutation via retrotransposition mechanism in a transient model on the HEK293 cell line: (**a**) Fluorescent microscopy of HEK293 cells transfected with a mix of prime editing vectors: mCherry^+^ cells contain edited *F9*-mCherry sequences with a restored reading frame. The upper row of images shows GFP fluorescence or transfection efficiency, while the lower row displays mCherry fluorescence or prime editing efficiency (magnification 50×); (**b**) Frequency of target editing according to the decomposition relative to control and reference sequence traces, * *p* < 0.05; (**c**) Frequency of indel mutations or incorrect prime editing in the target region of the *F9* gene, * *p* < 0.05.

To confirm the editing of *F9* mutations, the HEK293 cell line was transfected with a mix of substrate vector LV-*F9*-p.R379*-mCherry and editing constructs. The frequencies of mCherry^+^ cells were analyzed through microscopy 72 h after transfection (Figure 6a). The Padua and Shanghai mutations are known gain-of-function mutations of *F9* that were introduced into the RTT sequence to allow compensation for the low editing frequency by increasing the activity of FIX expressed from edited target alleles.

The results of subsequent genotyping summarizing data obtained from three biological and three technical replicates are presented in Figure 6b,c.

### 2.4. Prime Editing of the p.R379* Mutation in the F9 Gene via Retrotransposition Mechanism in a Transient Model on the Mouse MH-22a Cell Line

**Figure 7:**
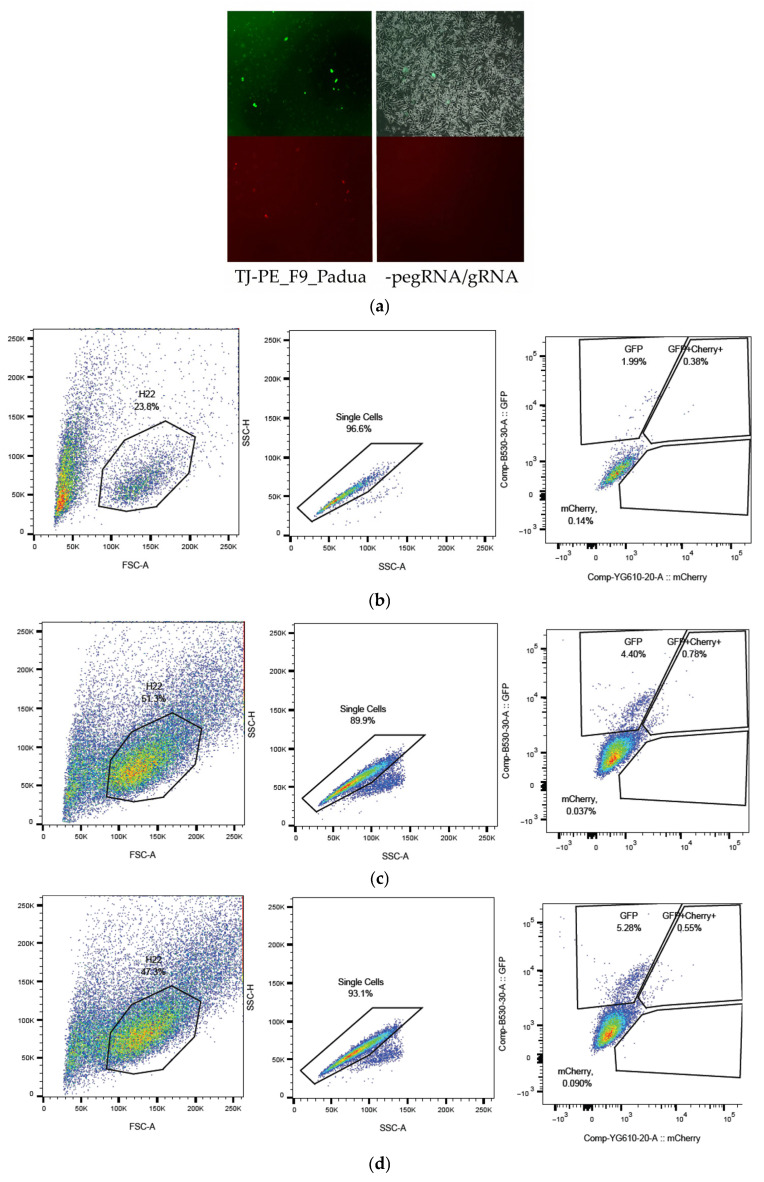
Editing of the *F9*_p.R379* mutation via retrotransposition mechanism in a transient model on the mouse hepatocarcinoma cell line MH-22a: (**a**) Fluorescent microscopy of MH-22a cells transfected with a mix of prime editing vectors pU6-TJ-100-Padua, B52-additional nicking gRNA-TJ-100-F9, and pCMV-PE2-SpRY-P2A-GFP (PE2): mCherry^+^ cells contain edited *F9*-mCherry sequences with a restored reading frame. The upper row of images displays GFP fluorescence or transfection efficiency, while the lower row shows mCherry fluorescence or prime editing efficiency (magnification 50×). The strategy for selecting and assessing the frequency of cells with a restored reading frame in *F9*-mCherry, indicative of successful prime editing of the *F9* p.R379* mutation, was conducted using flow cytometry: (**b**) without pegRNA, gRNA: 1.99% GFP^+^; GFP^+^mCherry^+^ and mCherry^+^ 0.55%; (**c**) *F9*_Padua_pegRNA, gRNA: 4.40% GFP^+^; GFP^+^mCherry^+^ and mCherry^+^ 0.82%; (**d**) *F9*_Shanghai_pegRNA, gRNA: 5.28% GFP^+^; GFP^+^mCherry^+^ and mCherry^+^ 0.64%.

To confirm the feasibility of editing via the retrotransposition mechanism using the same editing vectors, mouse *F9* sequences were employed in the mouse hepatocarcinoma cell line MH-22a. The frequencies of mCherry^+^ cells, reflecting the rate of successful editing of the substrate sequence *F9*_p.R379*-mCherry, were analyzed 72 h post-transfection (Figure 7).

**Figure 8:**
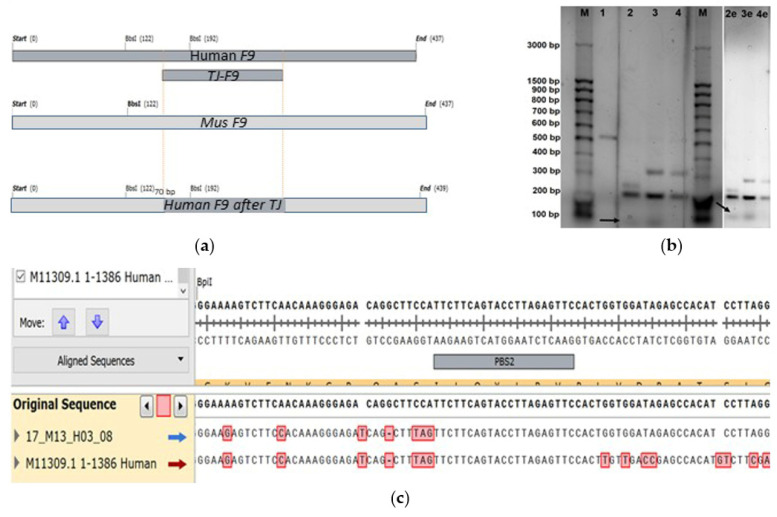
Comparison of PCR product sequences obtained on total DNA template after editing via retrotransposition mechanism, as well as non-edited control lines from human (HepG2) and mouse (MH-22a): (**a**) Structural maps of expected PCR products amplified from *F9* gene templates (top to bottom: human, mouse, edited chimeric sequence): the yellow dots indicate the homologous sequences of exon 8 of *F9* in human, mice, and the chimeric sequence; (**b**) Inverted images of the electrophoresis gel displaying the separation of PCR products hydrolyzed by BpiI obtained from the total DNA template after editing via retrotransposition (3), as well as non-edited control lines from human (HepG2 line: 1 for native PCR product, 2 for PCR products hydrolyzed by BpiI) and mouse (MH-22a line: 4 for PCR products hydrolyzed by BpiI of the *F9* fragment from mouse); M for DNA fragment length marker “100 bp + 1.5 Kb + 3 Kb” (SibEnz, Russia), with the bold arrow indicating the level of the 70 bp fragment corresponding to the edited chimeric sequence of mouse *F9* containing the human *F9* fragment inserted via retrotransposition; “e” indicates samples on the electrophoresis gel with increased image exposure; (**c**) Chromatogram of the chimeric sequence of the target fragment of the *F9* gene, resulting from prime editing via the retrotransposition mechanism with a frequency of 1%, as evidenced by Sanger sequencing of individual amplicons from the pool of PCR fragments of target *F9* sequences after editing on the MH-22a line, with the sequence aligned to the reference sequence of mouse *F9* alongside the control sequence of human *F9*: nucleotides that differ between human and mouse sequences are red marked.

Cells were collected for genotyping four days after transfection, and the target regions of the *F9* genes were amplified using total DNA (genomic and plasmid DNA from the substrate vector LV-*F9*-p.R379*-mCherry) as a template. The resulting heterogeneous amplicons were hydrolyzed with the restriction endonuclease BbsI, as the correct replacement of the gene fragment *MmF9* with the human *F9* gene sequence, flanked by two PBS, introduces a recognition site for the endonuclease BpiI. Upon hydrolysis, a fragment of 70 base pairs appears on the electrophoresis gel (Figure 8).

To determine nucleotide sequences, PCR products were ligated into the pAL-TA vector, and the insertions were sequenced using standard M13 primers. Analysis of the chromatograms revealed a 1% frequency of chimeric constructs arising from prime editing of the target region of the *F9* gene.

### 2.5. Assessment of FIX Factor Activity Changes Following Editing of the F9 p.R379* Mutation via Retrotransposition Mechanism in the HepG2 Cell Line

**Figure 9:**
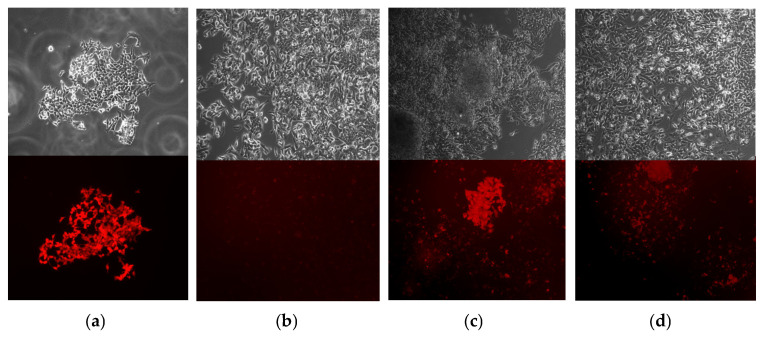
Editing of the stable line *F9*-p.R379*-mCherry: (**a**) Control cells KO-*F9*-HepG2 transduced with LV-*F9wt*-mCherry: top-phase-contrast microscopy, bottom-fluorescence microscopy in the mCherry detection channel (50× magnification); (**b**) KO-*F9*-HepG2 cells transduced with LV-*F9*-p.R379*-mCherry: top-phase-contrast microscopy, bottom-fluorescence microscopy in the mCherry detection channel (50× magnification); (**c**,**d**) Edited cells from the stable line HepG2-*F9*-p.R379*-mCherry: top-phase-contrast microscopy, bottom-fluorescence microscopy in the mCherry detection channel (50× magnification); mCherry^+^ cells contain substrate target sequences *F9*-mCherry with restored reading frame.

The next essential step in our study was to determine the significance of the conducted prime editing on the activity of the synthesized coagulation factor FIX in the stable lines HepG2_*F9*-p.R379* and p.R294*. The stable lines HepG2-*F9*-p.R379* and p.R294*, obtained after three rounds of transduction of the KO-*F9*-HepG2 line, were transfected with a mixture of editing vectors. After 96 h, the accumulation of fluorescent signal in the mCherry channel was observed, indicating the restoration of the reading frame of the target sequences *F9*-p.R379*-mCherry and *F9*-p.R294*-mCherry within the lentiviral inserts integrated into the genome of the recipient line (Figure 9).

**Figure 10:**
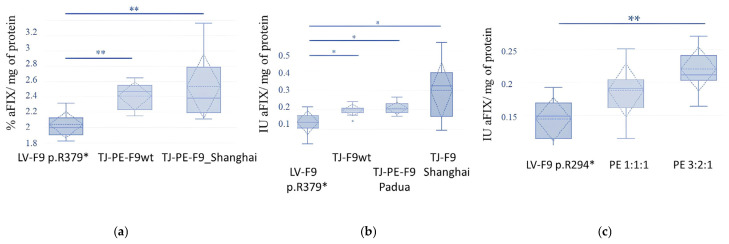
The impact of editing the p.R379* and p.R294* mutations on FIX Activity in stable HepG2 cell Lines: (**a**) Comparison of FIX activity in the supernatants of edited and control cell lines: standard deviation, confidence interval, medians, and means are indicated; statistical significance of the observed differences was assessed using the Kruskal-Wallis non-parametric test (*p* = 0.006), and confirmed by the Friedman test (*p* = 0.002). The Dunn–Bonferroni post-hoc test for pairwise comparisons between the groups LV-*F9*_p.R379*-LV-F9_p.R379*/TJ-PE-*F9*wt and LV-*F9*_p.R379*-LV-*F9*_p.R379*/TJ-PE-*F9*_Shanghai showed significant differences in each pair when summarizing data from three biological and three technical replicates (** Adj. *p* = 0.008), % aFIX (100%–1ME/mL); (**b**) Comparison of FIX activity in the concentrates of supernatants from edited and control cell lines: standard deviation, confidence interval, medians, and means are indicated; statistical significance of the observed differences in FIX activity between the edited and control cell pools was assessed using the Kruskal–Wallis non-parametric test (*p* = 0.031), and confirmed by the Friedman test (*p* = 0.006). The Dunn–Bonferroni post hoc test for pairwise comparisons between the groups LV-*F9*_p.R379*-LV-*F9*_p.R379*/TJ-PE-*F9*wt (*Adj. *p* = 0.015), LV-*F9*_p.R379*-LV-*F9*_p.R379*/TJ-PE-*F9*_Padua (Adj. * *p* = 0.047), and LV-F9_p.R379*-LV-F9_p.R379*/TJ-PE-F9_Shanghai (**Adj. *p* = 0.008) indicated significant differences in each pair when summarizing data from three biological and three technical replicates; (**c**) Comparison of FIX activity in the concentrates of supernatants from edited mixtures of editing vectors in two vector ratios (SpRY:pegRNA:gRNA, respectively) and control cell lines R294*: statistical significance of the observed differences in FIX activity between the edited and control cell pools was assessed using the Kruskal–Wallis non-parametric test (*p* = 0.003), and confirmed by the Friedman test (*p* = 0.002). The Dunn–Bonferroni post hoc test for pairwise comparisons showed significant differences for the groups LV-*F9*_p.R294* (3:2:1) and LV-*F9*_p.R294* (** Adj. *p* = 0.001).

HepG2-KO-p.R379* cells that were unedited but transfected with a reporter vector as part of the chimeric construct SpRY-GFP, as well as cells edited via the retrotransposition mechanism with the replacement of the target region of the *F9* gene with the wild-type sequence, and cells edited via the retrotransposition mechanism with the replacement of the target region of the *F9* gene with the *F9*_Padua/Shanghai sequence, were sorted and selected as GFP^+^ by flow cytometry. After 96 h of incubation, FIX activity was assessed in the supernatants of the selected transfected cells and their concentrates (Figure 10). Comparison of FIX activity in pools of edited and control cells revealed statistically significant differences according to two non-parametric criteria (adj. *p* = 0.008, Figure 10a).

Subsequently, the supernatants were concentrated tenfold, and a chromogenic assay was performed with normalization against total protein. The experiment confirmed the statistical significance of the observed differences in FIX activity in the supernatants of the edited cells compared to the unedited control cells (Figure 10b). The comparison of protein activity during prime editing of the stable line p.R294* demonstrated the advantage of using a vector ratio of 3:2:1 (SpRY, pegRNA, gRNA) (Figure 10c).

Thus, PE editing via the retrotransposition mechanism occurs with comparable efficiency to PE3 but has the advantage of universality and potential applicability for editing the region coding for amino acids 374 V–408 Q (numbering according to the human sequence), as demonstrated in the editing of one of the most common mutations, p.R379*, both in Russia (5%) and worldwide [15]. A distinct advantage of this gene therapy approach is the absence of the need to modify the PBS and RTT sequences when transitioning from DKI to KI, as well as the introduction of mutations to enhance the functions of chimeric FIX to mitigate low editing frequencies and enhance the therapeutic effect in vivo.

## 3. Discussion

Hemophilia B is one of the earliest known hereditary monogenic disorders associated with a deficiency of blood coagulation factor IX. The history of this disease dates back to ancient times, with its symptoms documented millennia ago. The story of hemophilia spans over 2000 years, and its connection to the royal families of Europe has facilitated its early study [16]. Despite the extensive history of observations and studies on the molecular and genetic consequences of Hemophilia B, as well as the primary triggers for its development, the underlying mechanisms now appear less obvious and more coincidental than previously thought. This paradox can largely be attributed to the complex structure of the protein, which includes two EGF-like domains, leading to pleiotropic effects of mutations in the *F9* gene, the severity of which may vary depending on the type and location of the mutation. It has been previously shown that the first EGF-like domain of blood coagulation factor IX weakens cellular adhesion and induces apoptosis via a caspase-3 dependent way [17]. Additionally, FIX has been identified as a crucial factor in the response to CDK4/6 inhibitor drugs, such as Palbociclib and Abemaciclib, which are employed to induce senescence in cancer cells. The loss of *F9* function obstructs this drug-induced senescence, highlighting the significant role of FIX in the therapeutic efficacy of these inhibitors. This interaction underscores the importance of understanding the molecular mechanisms underlying FIX’s involvement in cell cycle regulation and its potential implications for cancer treatment strategies [18].

It is well established that Cyclin D/CDK4 and Cyclin E/CDK2 are two pivotal protein complexes that regulate the transition of the cell cycle from the G1 phase to the S phase. These complexes operate sequentially: initially, Cyclin D/CDK4 phosphorylates the retinoblastoma protein (Rb), leading to the release of transcription factors such as E2F. This process subsequently promotes the production of Cyclin E, which activates the CDK2, facilitating the cell’s progression into the S phase. Therefore, Cyclin D/CDK4 is primarily responsible for the early stages of G1, governing the extended duration of this phase and preparing the cell for entry into S phase. However, the Cyclin E/CDK2 complex also plays a critical role in the transition from G1 to S phase, particularly during the later stages of G1. CDK2 interacts with Cyclin E to phosphorylate Rb at these later stages, further activating E2F and initiating DNA replication. Once activated by Cyclin E, this complex rapidly phosphorylates Rb to ensure that the cell fully commits to entering the S phase. The activation of Cyclin E/CDK2 occurs swiftly, generating a “burst” of activity essential for the G1/S transition. This coordinated action between the two complexes underscores the intricate regulatory mechanisms governing cell cycle progression [19]. CDK4/6 and CDK2 are both crucial regulators of cell proliferation, and the deletion of CDK4/6 can result in a reliance on CDK2 for continued cell cycle progression. This dependence on CDK2 presents a potential therapeutic opportunity, as the cell cycle remains active. Inhibiting CDK4/6 can lead to an increase in the levels of the cyclin-dependent kinase inhibitor p27, which subsequently inhibits CDK2, resulting in cell cycle arrest. Conversely, a complete deletion of CDK4/6 can allow cells to bypass the retinoblastoma (Rb) protein’s regulatory control, rendering them Rb-independent and activating CDK2. Therefore, targeting both pathways—CDK4/6 and CDK2—becomes essential for effective treatment strategies. By simultaneously inhibiting CDK4/6 and managing CDK2 activity, it may be possible to induce cell cycle arrest more effectively and prevent the proliferation of cancer cells that have adapted to evade conventional regulatory mechanisms. This dual-targeting approach could enhance therapeutic efficacy and improve outcomes in cancers characterized by dysregulated cell cycle progression [20].

In our study, we encountered the impact of extensive deletion of the *F9* gene on the proliferation of hepatocarcinoma cell cultures. Cells possessing the wild type allele of *F9* exhibited a proliferative and selective advantage, even within heterogeneous populations where they constituted a minor fraction among the majority of *F9* knockout cells. This advantage was observed even in the absence of CDK4/6 inhibitors, highlighting the significant role that the wild type *F9* allele plays in cell growth dynamics. The presence of the functional *F9* allele appears to confer a competitive edge, enabling these cells to thrive despite the predominance of genetically modified counterparts. This finding underscores the complex interplay between genetic alterations and cellular behavior in cancer biology, suggesting that restoring or enhancing the function of the *F9* gene may have therapeutic implications in managing hepatocarcinoma and potentially other malignancies [18]. It is established that Factor IX can interact with the kinase ERK, thereby influencing the activity of transcription factors within the ERK/MAPK signaling pathway as well as the serine protease KLK1. However, this interaction has only been studied as a result of large screening experiments and requires further detailed investigation [21,22]. Understanding the nature and implications of these interactions could provide valuable insights into the regulatory mechanisms involving FIX and its role in cellular processes, including proliferation and apoptosis. Further research is essential to elucidate the functional significance of these interactions and their potential impact on the pathophysiology of conditions such as hemophilia and cancer. Nevertheless, it is known that KRK1 treatment has been shown to increase the expression of Cyclin A and Cyclin E through the activation of the ERK1/2 signaling pathway in spermatogonial stem cells. This suggests that KRK1 may play a significant role in regulating cell cycle progression and proliferation in these cells by modulating key cyclins involved in the transition from G1 to S phase. Further studies are needed to explore the underlying mechanisms and the broader implications of this regulation in various cellular contexts [23]; therefore, it is likely that similar regulatory mechanisms exist in other human tissues, as well as the involvement of the coagulation factor FIX in the regulation of the eukaryotic cell cycle. New pathological types of mutations in the *F9* gene continue to be described with remarkable regularity, highlighting the ongoing need for research in this area to better understand the implications of these mutations on health and disease [3,24,25]. There are no so-called “hot spot” regions for mutations in the case of *F9*. Furthermore, alongside the documentation of a single mutation in a patient, other minor mutations may also be observed upon deeper sequencing of *F9* sequences. All of the above may indicate that the occurrence of mutations in the *F9* gene could be compensatory or adaptive in nature, aimed at mitigating adverse conditions or predispositions within the organism, such as uncontrollably dividing cells.

Based on the statistics of the etiology of hemophilia B both globally and in Russia, we have tested prime-editing approaches for the common mutations of *F9* associated with severe disease progression [3,15]. In this study, a TJ approach of *F9* prime-editing was successfully applied to the editing of one of the most frequently occurring mutations, p.R379*, both in Russia (5%) and worldwide.

## 4. Materials and Methods

### 4.1. Assembly of Editing Vectors for F9 Gene Knockout

To achieve a large deletion of the *F9* gene, we employed the CRISPR/Cas9 system within the editing vector PX458/pSpCas9(BB)-2A-GFP (Addgene #48138) [26]. Guide RNAs were designed to target exons 2 and 8 of *F9* using standard selection algorithms available in the online tool CRISPOR-Tefor [27]. The editing constructs were assembled via Golden Gate ligation of annealed and phosphorylated primers with linerised at BbsI (NEB, Ipswich, MA, USA) site vector PX458/pSpCas9(BB)-2A-GFP. The study utilized restriction endonucleases, T4 ligase, PNK, and SAP enzymes (NEB, Ipswich, MA, USA) according to the manufacturer’s recommendations. Vector amplification was performed through the transformation of competent cells from the *Escherichia coli* strain NEB-Stable (NEB, Ipswich, MA, USA). The sequences of the primers used are provided in Appendix A. The correctness of the assembly was confirmed by Sanger sequencing.

### 4.2. Assembly of Prime-Editing and Template-Jumping Vectors

The constructs for prime editing were assembled using the standard vectors pU6-pegRNA-GG-acceptor (Addgene #132777) for pegRNA assembly and B52 (Addgene #100708) for the expression of additional nicking gRNAs. PegRNAs were designed using the PE Design RGEN Tool algorithms [28]. Vector construction was carried out according to the recommended standard protocol previously published [19]. The pU6-pegRNA-GG-acceptor vector was digested with the restriction endonuclease BsaI-HF and dephosphorylated with rSAP phosphatase for 3 h. A 2.2 kb plasmid fragment (containing the replication origin, U6 promoter, U6 poly-T terminator, and AmpR sequences) was separated and purified from the digestion products using electrophoresis on a 1% agarose gel (TopVision agarose and TAE buffer, Thermo Fisher, Waltham, MA, USA) and the CleanUp Mini kit for DNA extraction from agarose gels (Evrogen, Moscow, Russia). The sequences of the primers used for pegRNA assembly are provided in Appendix A. The study utilized restriction endonucleases, T4 ligase, PNK, and SAP enzymes (NEB, Ipswich, MA, USA) according to the manufacturer’s recommendations.

The primer sequences for pegRNA assembly were composed as follows: the editing (target) sequence was included in the spacer primers and flanked by 5′ CACC and 3′ GTTTT sequences for the forward primer, and 5′ CTCTAAAAC for the reverse primer. To ensure effective transcription initiation, the spacer sequences began with a G-nucleotide. The template for reverse transcription was the “pegRNA 3′ extension template” flanked by 5′ GTGC for the forward sequence and 5′ AAAA for the reverse primer sequences. The sgRNA scaffold sequence contained complementary overlaps for assembly via the Golden Gate strategy, with the 5′ ends of the scaffold sequences phosphorylated by T4-PNK. The primers were renatured in pairs to obtain fragments for ligation in a buffer consisting of 10 mM Tris-Cl pH 8.5 and 50 mM NaCl, incubated at 95 °C for 3 min, followed by gradual cooling (0.1 °C/second) to 22 °C. Ligation with pU6-pegRNA-GG-acceptor/BsaI and rSAP was performed at a molar ratio of 1 µM for each of the three renatured fragments and 30 ng of vector, with the reaction carried out overnight at +4 °C. Amplification of the ligated vectors was achieved through the transformation of competent cells from the *E. coli* NEB-Stable strain. Following the screening of AmpR bacterial colonies and amplification of plasmids, the vectors were validated by restriction enzyme digestion and Sanger sequencing.

### 4.3. Transfection of HepG2 and MH-22a Cells

HepG2 cells (ICC RAS, passport №1-1-628, 02.09.2021) and MH-22a cells (ICC RAS, passport №1-1-631, 2021) were seeded in 24-well plates (Biofil, Shanghai, China) at a density of 1 × 10^5^ cells per well. Transfection was performed 16 h later in 250 μL of DMEM (Biosera, Beijing, China) without FBS (NeoFroxx, Eppelheim, Germany). After 4 h, the medium volume was adjusted to 500 μL, and replaced with fresh DMEM supplemented with 10% FBS after an additional 4 h. For transfection with the editing vectors PX458-g67 (GAGGTATAATTCAGGTAAATTGG) and PX458-g170 (CCTTAATCCAGTTGACAT-ACCGGG), plasmids were used at a 1:1 ratio, as previously described [26]. The pEGFP plasmid was used for control transfection [29].

Seventy-two hours post-transfection, cells were dissociated with 0.25% tryp-sin-EDTA and pelleted by centrifugation at 500× *g* for 5 min. The pellet was resus-pended in FACS buffer. All transfection conditions were performed in duplicate, with replicates pooled into a single sample during preparation. The percentage of GFP-positive cells was quantified by flow cytometry on a CytoFLEX instrument (Beckman Coulter, Brea, CA, USA). Optimal transfection conditions, balancing polyethylenimine (PEI, Polysciences, Warrington, PA, USA) cytotoxicity and transfection efficiency, were achieved using 500 ng of total DNA at a DNA:PEI mass ratio of 1:3 (per 1 × 10^5^ cells).

### 4.4. Isolation of GFP-Positive HepG2 Cells

HepG2 cells were seeded in 6-well plates (Biofil, Shanghai, China) at 5 × 10^5^ cells per well and transfected as described in Section 4.1. Seventy-two hours post-transfection, cells were prepared for sorting. The cells were trypsinized, pelleted, and resuspended in culture medium with 1% FBS. The suspension was filtered through a 45-μm cell strainer and sorted based on GFP fluorescence using a BD FACSAria III cell sorter (BD Biosciences, San Jose, CA, USA). Sorting gates were set using cells transfected with a reporter-less plasmid. The cells were sorted into high GFP and low GFP fractions.

Sorted GFP-positive cells were collected in 100% FBS and kept on ice to enhance viability and reduce the post-sort adaptation period. The cell suspensions were concentrated by centrifugation and resuspended in 150 μL of 100% FBS. The bulk (root population) was immediately transferred to a 96-well plate (Biofil, Shanghai, China), while the remaining cells were used for limiting dilution.

After a 4 h incubation in 100% FBS, 150 μL of FBS-free DMEM was added. The medium was replaced every two days following a PBS wash. Upon reaching 70–80% confluency, cells were passaged to larger culture surfaces. To minimize polyclonal line formation, cells from both high GFP and low GFP root populations (collected immediately after sorting and during passaging) were subjected to limiting dilution at a density of one cell per two wells of a 96-well plate (Biofil, Shanghai, China). Limiting dilution was performed using various cell populations, including those characterized by genotyping. Colony formation was monitored using a Zeiss AxioVert A1 (Carl Zeiss, Oberkochen, Germany) fluorescence microscope and the IncuCyte S3 system (Sartorius, Göttingen, Germany).

### 4.5. Genomic DNA Extraction and Genotyping

For genotyping, HepG2 cells were detached using 0.25% trypsin-EDTA, and pellets were collected by centrifugation at 300× *g* for 3 min. Then pellets were washed with DPBS (PanEco, Moscow, Russia), centrifuged, and stored at −80 °C. Genomic DNA was isolated using standard methods [30].

PCR was performed using the high-fidelity polymerase mix “Tersus Plus PCR kit” (Eurogen, Moscow, Russia). Two primer combinations were used: to detect the 25 kb deletion, primers *F9*_ex2_F (5′-cttgatcatgaaaacgccaac-3′) and *F9*_4seqR (5′-AGTGTGGTACTGGCCTTGTG-3′) were used, flanking a 539 bp DNA fragment in the case of a successful large deletion. Primers *f9* g172fw_IIF (5′-caccgGAATATATACCAAGGTATCC-3′) and *F9*_4seqR, yielding a 506 bp product, were used to detect the wild-type *F9* allele.

To decompose the results of Sanger sequencing and determine the frequencies of indels and prime editing, online algorithms from the TIDE [31] and ICE tools [32] were employed. The decomposition was performed relative to control chromatograms, comparing them with the reference sequence according to the HDR frequency assessment protocol. In the case of evaluating editing results during transient expression of the substrate vector, primers complementary to the target gene fragment of the *F9*-mCherry chimeric construct were used for amplification.

### 4.6. Lentivirus Production

Third-generation lentiviral vectors were produced in HEK293T cells by PEI-mediated co-transfection with four plasmids: the packaging plasmids pMDLg/pRRE, pRSV-Rev, pCMV-VSV-G, and the gene-of-interest (GOI) transfer plasmid (pGOI). The pGOI was constructed based on the LEGO-G plasmid and encoded, in place of GFP, one of the following *F9* cDNA variants: *F9*-mut379* (p.R379*), *F9*-mut294* (p.R294*), *F9*-mutR294L (p.R294L), *F9*-WT, or *F9*-Padua, followed by a T2A peptide and the mCherry fluorescent marker. Co-transfection with the LEGO-G plasmid served as a positive control.

Transfection was performed in 10 cm dishes (Nest, Suzhou, China). One hour before transfection, the medium was replaced with DMEM containing 2% FBS. The transfection mixture per dish contained 10 µg pMDLg/pRRE, 5 µg pRSV-Rev, 2 µg pCMV-VSV-G, and 20 µg pGOI at a DNA:PEI mass ratio of 1:3. The medium was changed 16 h post-transfection to DMEM supplemented with 10% FBS, L-glutamine (GlutaMAX, Gibco, New York, USA), and non-essential amino acids (PanEco, Moscow, Russia). Viral supernatants were collected 72 h post-transfection, centrifuged at 500× *g* for 5 min, and filtered through a 0.22-µm filter. The filtrate was either used directly for transduction or concentrated by centrifugation at 20,000× *g* for 2 h at 4 °C. The viral pellet was resuspended in DMEM, aliquoted, and stored at −80 °C. Transfection efficiency was monitored using a Zeiss AxioVert A1 (Carl Zeiss, Oberkochen, Germany) fluorescence microscope.

### 4.7. Lentivirus Titration

Lentiviral titer was determined by an end-point dilution assay on HEK293T cells (85120602, ECACC (European Collection of Authenticated Cell Cultures), 2024).

Cells were seeded in 24-well plates (Biofil, Shanghai, China) at 5 × 10^4^ cells per well one day prior to transduction. One hour before transduction, the medium was replaced with se-rum-free DMEM, and serial dilutions of the virus-containing medium were added to the wells. The medium was replaced with fresh DMEM containing 10% FBS 16 h later. Fluorescence was assessed at 48, 72, and 96 h using an IncuCyte S3 live-cell analysis system (Sartorius, Göttingen, Germany) and a Zeiss AxioVert A1 microscope (Carl Zeiss, Oberkochen, Germany). The titer, expressed as infectious units per milliliter (IU/mL), was calculated based on the percentage of mCherry-positive cells and the dilution factor.

### 4.8. Generation of HepG2 Cell Lines Expressing F9 cDNA Variants

HepG2 cells were seeded in 24-well plates (Biofil, Shanghai, China) at 5 × 10^4^ cells per well one day before transduction. Transduction was performed by culturing the cells in 20–100% virus-containing medium for 24 h in DMEM, with or without polybrene (10 µg/mL). Fluorescence was assessed at 48, 72, and 96 h post-transduction using a Zeiss AxioVert A1 (Carl Zeiss, Oberkochen, Germany) fluorescent microscope.

### 4.9. Transfection with TJ-Prime Editing Vectors

LV-HepG2-*F9*(p.R379*)-mCherry cells were transfected with vector cocktails for *F9* mutation reversion in a 12-well format (Biofil, Shanghai, China), with 10 technical replicates across 3 independent experimental groups. For prime editing of the *F9* gene (nonsense mutation p.R379*), three vector cocktails were used: (1) pU6-TJ-pegRNA-*F9*(WT)-SpRY, SpRY-GFP (Addgene #159981), nicking gRNA_*F9*; (2) pU6-TJ-pegRNA-*F9* (Padua)-SpRY, SpRY-GFP (Addgene #159981), nicking gRNA_*F9*; (3) pU6-TJ-pegRNA-*F9* (Shanghai)-SpRY, SpRY-GFP (Addgene #159981), nicking gRNA_*F9*. Editing plasmid mixtures were prepared at a 1:1 mass ratio. The SpRY-GFP vector (Addgene #159981) was included in all experiments at an equal DNA mass to control for transfection efficiency.

The cells were seeded in 12-well plates (Biofil, Shanghai, China) at 4 × 10^5^ cells per well in DMEM supplemented with 10% FBS, non-essential amino acids, L-glutamine, and incubated at 37 °C with 5% CO_2_. Transfection with editing construct mixtures was per-formed using PEI at 1 mg/mL. Two hours prior to transfection, cells were washed with DPBS and cultured in DMEM with 2% FBS. Plasmid DNA mixtures were combined with PEI at a 1:3 DNA: PEI ratio, targeting 600 ng total DNA per 1 × 10^5^ cells. The mixture was incubated for 13 min, and 100 μL (10% of the total medium volume) was added to the cells. Post-transfection, cells were incubated at 37 °C with 5% CO_2_. The medium was replaced 8 h later with DMEM containing 10% FBS, L-glutamine and non-essential amino acids. Transfection efficiency was assessed by fluorescence microscopy Zeiss AxioVert A1. Prime editing efficiency was determined by genotyping with locus-specific primers and Sanger sequencing (3500 Genetic Analyzer, Applied Biosystems, Foster City, CA, USA). Editing frequencies were quantified by flow cytometry followed by cell sorting on a BD FACSAria III platform.

### 4.10. Microscopy

Live-cell imaging was performed using a Zeiss Axio Vert A1 inverted fluorescence microscope. Cells were monitored at 24, 48, 72, and 96 h to assess cell state and confirm transfection efficiency. Phase-contrast imaging was used for general morphology and confluence assessment. GFP-positive cells were detected using a 488/509 nm filter set, and mCherry signal was detected using the RFP channel (587/610 nm). Images were acquired with ZEN 1.0 software using 5× and 10× objectives for both overview analysis of culture density and detailed examination of single-cell morphology.

Live-cell imaging with time-lapse acquisition of HepG2 knockout cells was per-formed using the Tomocube HT-X1 (Tomocube, Daejeon, Republic of Korea), a compact lens-free tomographic microscope enabling label-free volumetric reconstruction. Cells were imaged in GFP and brightfield channels at 40× magnification over a continuous 96 h period post-transfection to monitor cellular dynamics.

### 4.11. Flow Cytometry Sorting

Cells were sorted on day 5 post-transfection on a BD FACSAria III sorter. Instrument setup and gating used untransfected cells as a negative control. Cells were washed with DPBS, dissociated with 200 μL of trypsin-EDTA, and the reaction was stopped with complete medium. Cells were washed twice with DPBS and resuspended in sterile-filtered DPBS containing 1% FBS and HEPES. The suspension was filtered through a 40-μm cell strainer (Biologix, Xi’an, China, # 15-1040) to remove aggregates. The sorted cell fraction was collected into 15 mL tubes containing 100% FBS (+4 °C), plated in a 96-well plate (Biofil, China), and cultured under standard conditions. Upon reaching 80–90% confluency, cells were passaged to larger flasks.

### 4.12. FIX Activity Assays

HepG2 cells were seeded in 6-well plates (Biofil, China) at 5 × 10^5^ cells per well. The following day, the medium was replaced with fresh DMEM supplemented with 10% FBS, non-essential amino acids, L-glutamine, and 350 ng/mL Vitamin K3. Cells were incubated in a light-protected incubator at 37 °C for 96 h.

Supernatants were collected and centrifuged at 300× *g* or 3 min to remove cell de-bris. The supernatant was then centrifuged at 3000× *g* for 15 min to pellet fine cellular fragments. FIX protein was concentrated from the supernatant using 10 kDa molecular weight cut-off (MWCO) microcentrifuge filters (Biofil, China, # 110105) by centrifugation at 10,000× *g* for 8 min, achieving a 10-fold volume reduction.

The cell monolayer was washed twice with cold DPBS and lysed in 200 μL of RI-PA buffer supplemented with a 1× Roche protease inhibitor cocktail. Lysis proceeded on ice for 20 min, followed by centrifugation at 7000× *g* for 10 min to remove debris. Supernatant and lysate samples were stored at −20 °C for subsequent analysis. FIX activity in supernatants was determined using the Rox Factor IX chromogenic as-say kit (Rossix, Lund, Sweden, # 900020) according to the manufacturer’s instructions. Briefly, 10 μL of sample or standard was added to wells in quadruplicate. Calibration standards were prepared by serial dilution of a human plasma pool in the kit’s buffer to final dilutions of 1:40, 1:80, 1:160, 1:320, 1:640, 1:2000, and 1:4000. Then, 10 μL of reagent A was added to each well and incubated for 4 min at 37 °C, followed by the addition of 60 μL of reagent B and an 8 min incubation at 37 °C. Finally, 20 μL of substrate was added, and the plate was incubated for 4 min at 37 °C. Absorbance was measured at 490 nm with a reference wavelength of 630 nm using a CLARIOstar Plus multimode plate reader (BMG LABTECH, Ortenberg, Germany). FIX activity was calculated based on the standard curve.

Total protein in supernatants was quantified using the DC Protein Assay (Bio-Rad, Hercules, CA, USA, # 500-0112) for normalization of FIX activity. A volume of 5 μL of sample or standard (BSA, 0.1–10 mg/mL) was added to wells in quadruplicate. Then, 25 μL of reagent A’ and 200 μL of reagent B were added sequentially. The plate was incubated at 37 °C for 15 min, and absorbance was measured at 750 nm using the CLARIOstar Plus reader (BMG LABTECH, Ortenberg, Germany).

### 4.13. Western Blotting

Proteins were separated by SDS-PAGE using a Mini-PROTEAN Tetra Vertical Electrophoresis Cell (Bio-Rad, USA). A two-layer gel was used: 4% stacking gel and 10% resolving gel. Electrophoresis was performed in two stages. First, samples of concentrated supernatant and cell lysate were loaded and run at a constant voltage of 80 V for 20 min to focus the proteins in the stacking gel. Second, after the samples entered the resolving gel, the voltage was increased to 120 V and electrophoresis continued for 2 h for effective separation by molecular weight.

Proteins were transferred to a PVDF membrane (Millipore) using the wet transfer method in transfer buffer containing 10% methanol, at a constant voltage of 30 V for 16 h. The membrane was then washed three times for 10 min each with PBS containing 0.1% Tween-20 (PBST). To assess transfer efficiency and uniformity, the mem-brane was incubated with Ponceau S staining solution for 10 min. After documenting the stain, it was completely removed by washing three times with PBST.

For immunoblotting, the membrane was blocked with 5% non-fat dry milk in DPBS for 1–2 h at room temperature with gentle agitation (300 rpm) and washed again with PBST. The membrane was incubated with a primary Anti-Factor IX Anti-body (Huabio, San Diego, CA, USA, # HA500035) diluted 1:1000 in 5% milk at 4 °C overnight with constant agitation, followed by four washes with PBST. Subsequently, the membrane was incubated with an HRP-conjugated Anti-rabbit IgG secondary antibody (R&D Systems, Minneapolis, MN, USA, # AF008) for 2 h at room temperature with agitation. Unbound secondary antibody was removed by three washes with PBST.

The chemiluminescent signal was detected using an ECL reagent on a Chemi-Doc™ MP Imager (Bio-Rad, USA). The presence and molecular weight of FIX were determined by comparison with a protein marker (10–250 kDa range) and a positive control. Band intensities were analyzed for a comparative assessment of *F9* expression levels between cell lines.

### 4.14. Statistics

Statistical analyses of the data were performed using online tools: [33] and Excel tools with statistical add ins. The Kruskal–Wallis and Fisher’s non-parametric tests were used, the Dunn–Bonferroni post hoc test was used for pairwise comparisons [34,35,36].

## Data Availability

The original contributions presented in this study are included in the article/Supplementary Material. Further inquiries can be directed to the corresponding author.

## Supplementary Materials

The following supporting information can be downloaded at: https://www.mdpi.com/article/10.3390/ijms262411916/s1.

## Appendix A

**Table A1:** Nucleotide sequences of primers.

Name	Sequence (5′-3′)	Purpose
f9 g67fw_IF	caccgGAGGTATAATTCAGGTAAAT	Editing vectors for KO-F9 HepG2
f9 g67fw_IR	aaacATTTACCTGAATTATACCTCc	
f9 g170rev_IF	caccgCTTAATCCAGTTGACATACC	
f9 g170rev_IR	aaacGGTATGTCAACTGGATTAAGc	
f9 g106fw_IIF	caccgGAACCTTGAGAGAGAATGTA	
f9 g106fw_IIR	aaacTACATTCTCTCTCAAGGTTCc	
f9 g172fw_IIF	caccgGAATATATACCAAGGTATCC	
f9 g172fw_IIR	aaacGGATACCTTGGTATATATTCc	
F9_ex2_F	CTTGATCATGAAAACGCCAAC	F9 genotyping
F9_ex8_R	GTGAGCTTTGTTTTTTCCTTAATCCAG	
F9_881_spacer oligo (up)	CACC GCGCAGGTGAACATAATATTG GTTTT	Assembly of PE2 editing vectors
F9_881_spacer oligo (down)	CTCTAAAAC CAATATTATGTTCACCTGCGC	
F9_881_3’ extension oligo (up)	GTGC TTTCGCTTTTGCTCTGTATGTTCTGTCTCCTCAA TATTATGTTCACCTGCG	
F9_881_3’ extension oligo (down)	AAAA CGCAGGTGAACATAATA TTGAGGAGACAGAACATACAGAGCAAAAGCGAAA	
F9_881_additional nicking spacer oligo (up)	CACC GGTTCCAGAAGGGCAATGTCA	
F9_881_additional nicking spacer oligo (down)	AAAC TGACATTGCCCTTCTGGAACC	
pegRNA scaffold F	AGAGCTAGAAATAGCAAGTTAAAATAAGGCTAGTCCGTTATCAACTTGAAAAAGTGGCACCGAGTCG	
pegRNA scaffold R	GCACCGACTCGGTGCCACTTTTTCAAGTTGATAACGGACTAGCCTTATTTTAACTTGCTATTTCTAG	
mCherry_RI_R	GACGAATTCTTACTTGTACAGCTCGTCCATGCC	Assembly of chimeric sequences *F9*-mCherry
Hb (R294*) Oligo 4 (Insert 2) R	CGCGCTGCATAGGCCCGGGGTTTTCTTCAAC	
HB (R294*)Oligo 5 (Insert 3) F	CCCCGGGCCTATGCAGCGCGTGAACATGA	
HB (R294*) Oligo 6 (Insert 3) R	CTCCCGATCCTAAGTGAGCTTTGTTTTTTCCTT	
HB (R294*) Oligo 7 (Insert 4) F	AGCTCACTTAGGATCGGGAGAGGGCAGAG	
F9(R294*)PE2-SpCas9_spacer oligo (up)	CACCGGCTGCATTGTAGTTGTGGTGGTTTT	Assembly of PE2 editing vectors
F9(R294*)PE2-SpCas9_spacer oligo (down)	CTCTAAAACCACCACAACTACAATGCAGCC	
F9(R294*)PE2-SpCas9_3’ extension oligo (up)	GTGCAGCGAAATGTGATTCGAATTATTCCTCACCACAACTACAATGC	
F9(R294*)PE2-SpCas9_3’ extension oligo (down)	AAAAGCATTGTAGTTGTGGTGAGGAATAATTCGAATCACATTTCGCT	
F9(R294*)PE2-SpCas9_additional nicking spacer oligo (up)	CACCGCGCAGGTGAACATAATATTG	Assembly of a vector for expression of additional nicking RNA
F9(R294*)PE2-SpCas9_additional nicking spacer oligo (down)	AAACCAATATTATGTTCACCTGCGC	
F9(R294*)_PE2-SpRY_spacer oligo (up)	CACCGTGAGGAGACAGAACATACAGGTTTT	Assembly of PE2 editing vectors
F9(R294*)_PE2-SpRY_spacer oligo (down)	CTCTAAAACCTGTATGTTCTGTCTCCTCAC	
F9(R294*)_PE2-SpRY_3’ extension oligo (up)	GTGCTCGCTTTTGCTCTGTATGTTCTGTCTCC	
F9(R294*)_PE2-SpRY_3’ extension oligo (down)	AAAAGGAGACAGAACATACAGAGCAAAAGCGA	
F9(R294*)_PE2-SpRY_additional nicking spacer oligo (up)	CACCGAGTGGGCAGCAGTTACAATC	Assembly of a vector for expression of additional nicking RNA
F9(R294*)_PE2-SpRY_additional nicking spacer oligo (down)	AAACGATTGTAACTGCTGCCCACTC	
F9(R294L)_PE2-SpRY_spacer oligo (up)	CACCGGACAGAACATACAGAGCAAAGTTTT	Assembly of PE2 editing vectors
F9(R294L)_PE2-SpRY_spacer oligo (down)	CTCTAAAACTTTGCTCTGTATGTTCTGTCC	
F9(R294L)_PE2-SpRY_3’ extension oligo (up)	GTGCTCGAATCACATTTCGCTTTTGCTCTGTATGTTCT	
F9(R294L)_PE2-SpRY_3’ extension oligo (down)	AAAAAGAACATACAGAGCAAAAGCGAAATGTGATTCGA	
F9(R294L)_PE2-SpRY_additional nicking spacer oligo (up)	CACCGGTTTCAACACAGTGGGCAGC	Assembly of a vector for expression of additional nicking RNA
F9(R294L)_PE2-SpRY_additional nicking spacer oligo (down)	AAACGCTGCCCACTGTGTTGAAACC	
F9_C1135T (R379*) F	TTGTTGAC**TGA**GCCACATGTCTTCGATCTACA	*F9*-mutagenesis
F9_C1135T (R379*) R	TGGC**TCA**GTCAACAAGTGGAACTCTAAGGT	
F9_C880T (R294*)F	AGCAAAAG**TGA**AATGTGATTCGAATTATTCCTCA	
F9_C880T (R294*)R	CATT**TCA**CTTTTGCTCTGTATGTTCTGTCTCC	
F9_G881T (R294L)F	AGCAAAAG**CTA**AATGTGATTCGAATTATTCCTCA	
F9_G881T (R294L)R	CATT**TAG**CTTTTGCTCTGTATGTTCTGTCTCC	
TJ_F9nicking spacer oligo (up)	CACCGGGGTCCCCCACTATCTCCTT	Assembly of TJ-PE editing vectors
TJ_F9nicking spacer oligo (down)	AAACAAGGAGATAGTGGGGGACCCC	
TJ_F9spacer oligo (up)	CACCGTCTTCAGTACCTTAGAGTTCGTTTT	
TJ_F9spacer oligo (down)	CTCTAAAACGAACTCTAAGGTACTGAAGAC	
TJ-pegRNA scaffold F	AGAGCTAGAAATAGCAAGTTAAAATAAGGCTAGTCCGTTATCAACTTGAAAAAGTGGGACCGAGTCG	
TJ-pegRNA scaffold R	GGACCGACTCGGTCCCACTTTTTCAAGTTGATAACGGACTAGCCTTATTTTAACTTGCTATTTCTAG	
RT-PBS2_F	GTCCGCCCCCACTATCTC	
RT-PBS2_R	CAAGGAGATAGTGGGGGC	
F9-INS1_F	CTTGACATGAATCTCTACCTCCTTCATG	
F9-INS1_R	CTTCCATGAAGGAGGTAGAGATTCATGT	
F9-INS2_F	GAAGCCAGCACAGAACATGTTGTTATAG	
F9-INS2_R	CCATCTATAACAACATGTTCTGTGCTGG	
F9-INS3wt_F	ATGGTGAACTTTGTAGATCGAAGACATGTG	
F9-INS3wt_R	GAGCCACATGTCTTCGATCTACAAAGTTCA	
F9-INS4_PBS1_F	GCTCGGTCAACAAGTGGAACTCTAAG	
F9-INS4_PBS1_R	GTACCTTAGAGTTCCACTTGTTGACC	
F9-INS5_PBS1_F	GTACTGATCTCTCTCTTGACGCG	
F9-INS5_PBS1_R	GAACCGCGTCAAGAGAGAGATCA	
F9-INS6_Tevopreq1_F	GTTCTATCTAGTTACGCGTTAAACCAACTAGAAA	
F9-INS6_Tevopreq1_R	AAAATTTCTAGTTGGTTTAACGCGTAACTAGATA	
F9-INS3_P_F	ATGGTGAACTTTGTAGACAGAAGACATGTG	
F9-INS3_P_R	GAGCCACATGTCTTCTGTCTACAAAGTTCA	
F9-INS3_Sh_F	ATGGTGAACTTTGTAGACTGAAGACATGTG	
F9-INS3_Sh_R	GAGCCACATGTCTTCAGTCTACAAAGTTCA	
